# Immunosenescence-related T cell phenotypes, structural brain imaging, and cognitive impairment in patients with schizophrenia: a moderated mediation analysis

**DOI:** 10.1038/s41537-025-00650-w

**Published:** 2025-07-18

**Authors:** Na Li, Yanli Li, Ting Yu, Wenjin Chen, Mengzhuang Gou, Wenkai Zheng, Zhaofan Liu, Xiaoying Wang, Jiao Fang, Jinghui Tong, Song Chen, Baopeng Tian, Chiang-Shan R. Li, Li Tian, Yunlong Tan

**Affiliations:** 1 Peking University HuiLongGuan Clinical Medical School, Beijing HuiLongGuan Hospital, Beijing, P. R. China; 2 School of Basic Medicine, Inner Mongolia Medical University, Hohhot, China; 3 Department of Psychiatry, Yale University School of Medicine, New Haven, CT USA; 4 Department of Psychology and Logopedics, Faculty of Medicine University of Helsinki, Helsinki, Finland

**Keywords:** Neuroscience, Biomarkers

## Abstract

Cognitive impairment is a core characteristic of schizophrenia. Immunosenescence has been consistently implicated in the cognitive dysfunction observed in neurodegenerative diseases, but how it may relate to cognitive deficits in schizophrenia is still unclear. We explored the associations between immunosenescence and cognitive impairment in patients with schizophrenia (SCZ, *n* = 65) and healthy controls (HCs, *n* = 39). Immunosenescence markers were assessed by flow cytometry and included the percentage of naïve or memory T cell subsets labeled by CD4+/CD8+, CD45RA+(naïve)/CD45RO (memory), or CD95+(memory), as well as the intracellular levels of selected cytokines (IL-1β, IL-6, TNF-α, and IFN-γ) in T cell subsets. T1-weighted magnetic resonance imaging was performed to assess the subcortical volume and cortical thickness. Participants were evaluated using the Positive and Negative Syndrome Scale and the Chinese version of the MATRICS Consensus Cognitive Battery.The results indicated that (1) Compared with HCs, SCZ patients were characterized by fewer naïve and more memory T cell subsets, accompanied by altered intracellular cytokine levels, indicating immunosenescence phenotypes. (2) The intracellular IL-1β level in naïve CD8+CD45RA+CD95+ T cells was associated with working memory deficit in SCZ patients. (3) In a moderated mediation model, the effect of the IL-1β level on the working memory score was mediated by the thickness of the right inferior parietal lobule (IPL_R), and the volume of the right choroid plexus (CP) moderated the indirect pathway between the IL-1β level and IPL_R thickness. Our findings highlighted immunosenescence-related T cell phenotypes and the CP as potential biomarkers of cognitive deficit in SCZ.

## Introduction

Patients with schizophrenia experience a reduced life expectancy by 13–15 years and substantial cognitive deficits^1^, which likely reflect premature or accelerated aging^2^. Immunosenescence is considered a key mechanism underlying cognitive decline in neurodegenerative diseases^3^. However, its involvement in the cognitive deficit observed in schizophrenia remains unclear.

In immunosenescence, the production of naïve T cells decreases, while that of highly differentiated, antigen-exposed memory T cells increases^4^. These alterations have been found to be more pronounced in CD8 + T cells than in CD4 + T cells^5^. The loss of CD45RA expression and gain of CD45RO expression with increasing age are hallmarks of senescent T cells^6^. Apoptosis remodeling is another critical component of T cell senescence, as manifested by the low or absent CD95 (APO-1/Fas) cell surface expression on peripheral naïve T cells and relatively high levels of CD95 expression in activated memory T cells in the elderly^7^. Other than alterations in cell counts, in terms of functional impairment, naïve and memory T cells are characterized by the manifestation of the senescence-associated secretory phenotype (SASP) during aging. This includes abnormal levels of pro-inflammatory cytokines, such as interleukin (IL)-1β, tumor necrosis factor-α (TNF-α), IL-6, and other pro-inflammatory markers that modify the extracellular environment^8,9^.

Numerous studies have shown cognitive impairment is correlated with lower numbers of naïve T cells and higher numbers of memory T cells in neurotypical populations, as well as in elderly individuals with impaired cognition, including those with Alzheimer’s disease^3,10^. Until recently, only few studies have focused on T cell subsets with single CD45 or CD95 marker in schizophrenia patients. For example, one study reported increased levels of CD4 + 45RA in schizophrenia patients^11^. Additionally, the monocyte-induced microglia-like phenotypes found in schizophrenia patients are characterized by higher CD95 expression in patients with schizophrenia than in healthy controls^12^, while the increased serum CD95 levels of schizophrenia patients are independent of symptomatology^13^. Notably, neither single CD45RA- nor CD95 + T cells can be considered truly senescent^14^. In this study, we aimed to detect the distribution of CD4 + /CD8 + T cells double-labeled by CD45 and CD95 to identify a potential fully senescent T cell population.

The SASP of senescent T cells, particularly age-related cytokine dysregulation, is also involved in cognitive impairment. Glia excitation induced by overexpression of pro-inflammatory molecules (e.g., TNF-α) significantly contributes to cognitive decline^15^. Both IL-1 and IL-6 affect cognitive function by regulating neurogenesis and synaptic plasticity, such as via long-term potentiation^16,17^. Further, in rodent models of aging, IFN-γ was shown to influence the hippocampal structure, neuronal morphology, and synaptic plasticity, as well as cell density, thus influencing learning and memory^18^.

These immunosenescent T cell phenotypes may contribute to cognitive impairment in patients with schizophrenia by affecting the structure and function of the central nervous system (CNS). The choroid plexus (CP), the principal structure of the blood–CSF barrier and a vital source of CSF production, can function as a physical, enzymatic, and immunological barrier in circulation–CNS interfaces^19^. The CP has long been recognized as a gateway regulating the interactions between peripheral immune activation and brain dysfunction^20^. Upregulation of immune genes in the CP, inhibition of CP gateway function by IL-1β and TNF-α, and increased CP volumes have been described in individuals with schizophrenia^21,22^. Additionally, an association between poorer cognition and enlargement of the CP has been identified in schizophrenia patients^19^. Therefore, this study focused on the role of CP in linking immunosenescence to cognitive impairment in schizophrenia patients. Furthermore, different cognitive domains in schizophrenia may exhibit complex and region-specific neuroimaging characteristics. For example, the prefrontal cortex, parietal cortex, and hippocampus have been identified as critical regions for working memory performance^23^. Specifically, the inferior parietal lobule (IPL) serves as a cross-modal hub for integrating sensory inputs and maintaining task-relevant information^24^. In schizophrenia patients, structural and functional abnormalities in the IPL could contribute to working memory deficits^25^. Thus, we hypothesize that immunosenescence-related T cell phenotypes may exert effects on cognitive impairment across different dimensions by impacting different brain regions.

Based on the existing literature, we hypothesize that schizophrenia (SCZ) patients may demonstrate more significant immunosenescence-related T phenotypes compared to healthy controls (HCs). To better recognize naïve and senescent T cell subsets, we examined T cell subsets that were labeled with CD4/CD8, CD45, or CD95, either singly, doubly, or triply. We propose that alterations in the number and function of naïve and memory T cell subsets may serve as sensitive biomarkers associated with structural brain abnormalities and cognitive symptoms in SCZ. Most importantly, CP may play a critical role in linking peripheral immunosenescence, structural brain parenchymal abnormalities, and cognitive impairment in patients with SCZ.

## Materials and methods

### Participants

This study was approved by the Institutional Ethics Committee of Beijing Huilongguan Hospital and was conducted under the ethical principles outlined in the Declaration of Helsinki. Written informed consent was obtained from all participants before enrollment.

The SCZ group (*n* = 65) was recruited from Beijing Huilongguan Hospital, Beijing, China. The inclusion criteria were as follows: (1) Diagnosis of SCZ according to the Statistical Manual of Mental Disorders IV (DSM-IV); (2) aged 18–60 years old, Han nationality, right handedness, and > 6 years of education; (3) illness duration ≥5 years; (4) stable antipsychotic medication for at least 1 year before inclusion, and use of the chlorpromazine equivalent (CPZ) to standardize the dosage of antipsychotic medications. The HC group (*n* = 39) was recruited from a local community by advertisement. The age, sex, and years of education were comparable between the HC and SCZ groups.

The exclusion criteria for all participants were as follows: (1) DSM-IV Axis-II and other DSM-IV Axis I diagnoses; (2) Current or historical diagnoses of major physical diseases (e.g., nervous system diseases, cerebrovascular disease, diabetes, or cancer); (3) allergies requiring immunosuppressive therapy or chronic allergies with active symptoms, autoimmune disorder, chronic inflammation, and use of anti-inflammatory agents, immune-suppressive agents, or immune modulators in the previous 3 months; (4) current (within the past 12 months) or historical substance use disorders (per DSM-IV criteria), except for tobacco use or occasional alcohol consumption; and (5) pregnant or breastfeeding.

### Clinical assessments

Cognitive performance was evaluated using the Chinese version of the MATRICS Consensus Cognitive Battery (MCCB) test^26^. The seven dimensions of the MCCB test included: attention/vigilance, processing speed, working memory, visual learning, verbal learning, reasoning and problem-solving, and social cognition. The psychotic symptoms were evaluated using the Positive and Negative Syndrome Scale (PANSS), which includes positive, negative, and general subscale scores. The interviews were conducted by two independent psychiatrists, and the intraclass correlation coefficient was ≥ 0.80.

### Immunophenotyping

In the flow cytometric staining procedure, we examined the percentage of T cell subpopulations among the total T cell population, including CD4+ and CD8 + T cell subsets; naïve T cell subsets, including CD4 + CD45RA + , CD4 + CD45RA + CD95 + , CD8 + CD45RA + , and CD8 + CD45RA + CD95 + ; and memory T cell subsets, including CD4 + CD45RO + , CD8 + CD45RO + , CD4 + CD95 + , CD8 + CD95 + , CD4 + CD45RO + CD95 + , and CD8 + CD45RO + CD95 + . Since CD8 + T cells acquire an immunosenescent phenotype more quickly and more pronouncedly than CD4 + T cells^5^, the levels of intracellular cytokines, including TNF-α, IFN-γ, IL-1β, and IL-6, within the CD8 + CD45RA + CD95+ and CD8 + CD45RO + CD95 + T cell subsets were further examined. The measurement procedure and gating strategy have been described in detail previously^27^ and in the supplemental material.

### Structural magnetic resonance imaging (MRI) data acquisition and preprocessing

The T1-weighted high resolution structural brain imaging data were acquired using a 3.0 T MRI scanner (Siemens Medical Solutions, Germany) equipped with a 64-channel head coil at the Imaging Research Center of Beijing Huilongguan Hospital following published routines^28^.Specifically, FreeSurfer v6.0 (https://surfer.nmr.mgh.harvard.edu/) was used to acquire ventricle and choroid plexus (CP) volumes, seven regional subcortical volumes per hemisphere, thirty-four regional cortical thicknesses (based on the Desikan/Kiliani atlas) per hemisphere, and total intracranial volume (ICV). The MRI scanning, scanner parameters, and operational details are described in the supplemental file.

### Statistical analysis

SPSS V25.0 (IBM Corp., Armonk, NY, USA) was used for statistical analyses. The normal distribution of the data was tested using Shapiro–Wilk Normality Test. For variables that deviated from a normal distribution, sensitivity analyses using nonparametric methods (Mann–Whitney U test and Spearman correlation analysis) were additionally conducted to verify the robustness of findings. In the analysis for demographic and clinical measures, the χ2 test was performed for categorical variables, the independent *t*test was used for continuous variables. The analysis of covariance (ANCOVA) was applied to compare the cognitive performance (seven subscale scores and the total score of MCCB), immunosenescence markers (T cell subpopulation percentages and intracellular cytokine levels), and neuroimaging measures (ventricular volumes, bilateral CP volumes, seven subcortical volumes and thirty-four cortical thickness per hemisphere) between groups. Furthermore, Cohen’s *d* was calculated to evaluate the effect sizes of group differences. Pearson’s partial correlation analysis and hierarchical regression analysis were performed to assess the associations between immunosenescence, brain imaging, and clinical measures before conduction of mediating and moderating effects. Mediation examines how or why an independent variable (X) influences a dependent variable (Y) through an intermediate mechanism (M), testing whether X affects Y indirectly via M (i.e., X → M → Y)^29^. Moderation investigates under what conditions or for whom the X-Y, X-M, or M-Y relationships vary in strength/direction^29^. In this study, both approaches were used to integrate mechanistic explanations with boundary conditions, providing a more comprehensive understanding of the complex relationships among variables^29^. PROCESS v4.1 for SPSS was applied to test the hypotheses, with immunosenescence measurements as X, clinical measurements as Y, and structural imaging measurements as M (mediator) and W (moderator). Levels of the moderator variable were defined as low, medium, and high following the default protocol, corresponding to the 16th percentile (low), 50th percentile (median, medium), and 84th percentile (high) of the distribution^29^. The 95% confidence intervals (*CIs*) obtained from 5000 bootstrapped samples were calculated to evaluate the significance of the models. The Mediation and Moderated Mediation were tested via bias-corrected bootstrapping (5000 resamples), ensuring robustness to non-normality^29^. Sex was included as a covariate in all models. The subjects in our sample ranged in age from 18 to 60 years, and age was used as a covariate in all models. The ICV was added as a covariate in imaging data analysis. Educational level was a covariate in analysis for cognitive performance and imaging data. High-sensitivity C-reactive protein (h-CRP) was incorporated as a covariate in immunosenescence marker analyses due to its significant correlations with CD4+ (*r* = 0.325, *p* = 0.038) and CD8 + T cell percentages (*r* = −0.302, *p* = 0.045). The SCZ-specific covariates, such as age of psychosis onset, illness duration, and CPZ, were included in the correlation, regression, and mediation analyses for the SCZ group. Particularly, in hierarchical regression framework, core covariates (age, ICV, educational level, sex, body mass index [BMI], high-sensitivity C-reactive protein [hCRP]) were first, and SCZ-specific variables (age of psychosis onset, illness duration, and CPZ) were subsequently added. We also assessed the effect of psychopharmacological agents. Post hoc multiple analyses were performed using the Benjamini–Hochberg (BH) false discovery rate (FDR) method, and significance was defined as adjusted *p* value < 0.05^30^.

## Results

### Demographic and clinical characteristics

The demographic characteristics shown in Table 1 did not differ significantly between the SCZ and HC groups. The seven domains and composite T-scores of the MCCB were lower in the SCZ group than in the HCs (Table 2).

**Table 1:** Demographic and clinical details.

	SCZ (*n* = 65)	HC (*n* = 39)	*χ*^*2*^*/t*	*p*
Biological sex (M/F)	38/27	22/17	0.042	0.838
Age (years)	53.04 ± 7.87	50.97 ± 6.28	–1.397	0.166
Education (years)	11.58 ± 2.83	11.03 ± 3.67	–0.871	0.386
BMI (kg/m^2^)	24.66 ± 4.07	24.40 ± 2.85	–0.325	0.746
h-CRP (mg/L)	1.67 ± 1.41	1.94 ± 2.41	0.669	0.505
Age of psychosis onset (years)	23.57 ± 7.91	–	–	–
Duration (years)	29.62 ± 9.97	–	–	–
CPZ (mg)	529.75 ± 305.12	–	–	–
Clozapine dosage (mg)	182.30 ± 133.21	–	–	–
PANSS score				
Total	66.22 ± 11.97	–	–	–
Positive	15.70 ± 6.08	–	–	–
Negative	24.63 ± 6.17	–	–	–
General	25.88 ± 6.02	–	–	–

The demographic and clinical measures were not significantly different between the SCZ and HC groups. *BMI* body mass index, *h-CRP* high-sensitivity C-reactive protein, *CPZ* chlorpromazine equivalent, *PANSS* Positive and Negative Syndrome Scale, *SCZ* schizophrenia, *HC* healthy control.

**Table 2:** MCCB scores.

MCCB	SCZ	HC	*F*	*p*_*FDR*_	*Cohen’s d*
Speed of processing	43.71 ± 15.61	55.49 ± 10.76	15.188	2.427 × 10^–4^	**–0.88**
Attention/vigilance	34.33 ± 10.68	47.72 ± 10.80	34.121	3.476 × 10^–7^	**–1.25**
Verbal learning	44.88 ± 16.26	54.15 ± 10.07	9.355	0.003	**–0.69**
Visual learning	40.81 ± 11.86	50.03 ± 13.92	11.626	0.001	**–0.71**
Working memory	37.62 ± 15.31	54.05 ± 12.71	28.714	1.536 × 10^–6^	**–1.17**
Reasoning problem solving	40.98 ± 11.99	54.15 ± 10.07	28.073	1.536 × 10^–6^	**–1.19**
Social cognition	39.67 ± 11.78	51.23 ± 11.17	21.592	1.760 × 10^–5^	**–1.00**
Total score	37.16 ± 13.08	53.15 ± 12.53	33.752	3.476 × 10^–7^	**–1.25**

The seven domain T-scores and composite T-score of MCCB were lower in patients with SCZ than in HCs. The *p*_*FDR*_ was adjusted *p* value corrected for FDR correction.

*MCCB* MATRICS Consensus Cognitive Battery test, *SCZ* schizophrenia, *HC* healthy control, *FDR* false discovery rate correction.

### Flow cytometric analysis of T cell subpopulations and cytokines

**Fig. 1:**
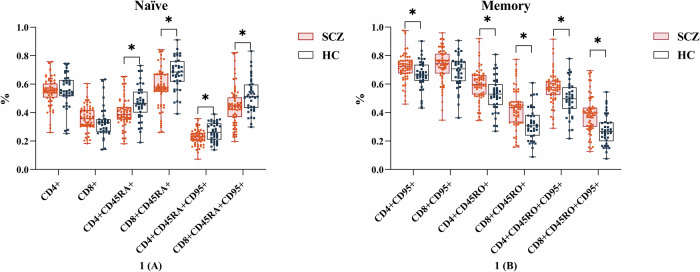
Comparison of the percentage of naïve and memory T cell subsets. **A** The comparison of the percentage of naïve T cell subsets between SCZ and HC groups. **B** The comparison of the percentage of memory T cell subsets between SCZ and HC groups. *p < 0.5, **p < 0.05. SCZ schizophrenia, HC healthy control.

The percentages of naïve T cell subpopulations, including CD4 + CD45RA + , CD8 + CD45RA + , CD4 + CD45RA + CD95 + , and CD8 + CD45RA + CD95 + T cells, were lower in the SCZ group than in the HCs; in contrast, the percentages of memory T cell subpopulations, including CD4 + CD45RO + , CD4 + CD95 + , CD4 + CD45RO + CD95 + , CD8 + CD45RO + , and CD8 + CD45RO + CD95 + T cell subsets, were higher in the SCZ group than in the HCs (Table 3; Fig. 1). The level of IL-1β in naïve CD8 + CD45RA + CD95+ and memory CD8 + CD45RO + CD95 + T cell subsets, as well as the level of IL-6, TNF-α derived from CD8 + CD45RO + CD95 + T cell subpopulations, were higher in the SCZ group than in the HCs. However, the levels of other cytokines did not differ significantly between the groups (Table 4).

**Table 3:** Differences in T cell composition between the SCZ and HC groups.

%	SCZ	HC	*F*	*p*_*FDR*_	*Cohen’s d*
CD4+ cells %					
CD4+	56.08 ± 13.52	53.78 ± 11.86	0.614	0.436	0.18
CD4 + CD45RA+	39.94 ± 14.33	47.09 ± 13.44	3.578	**0.026**	**–0.51**
CD4 + CD45RO+	59.97 ± 14.28	52.89 ± 13.44	3.449	**0.026**	**0.51**
CD4 + CD95+	72.22 ± 11.68	66.69 ± 11.45	4.173	**0.026**	**0.48**
CD4 + CD45RA + CD95+	22.64 ± 6.65	26.65 ± 7.16	3.495	**0.026**	**–0.58**
CD4 + CD45RO + CD95+	57.41 ± 14.19	49.83 ± 13.69	3.817	**0.026**	**0.54**
CD8+ cells %					
CD8+	36.43 ± 10.15	33.85 ± 12.85	1.350	0.291	0.22
CD8 + CD45RA+	57.25 ± 16.99	67.83 ± 12.80	8.359	**0.017**	**–0.70**
CD8 + CD45RO+	42.65 ± 17.03	32.15 ± 12.80	7.544	**0.018**	**0.70**
CD8 + CD95+	72.90 ± 13.97	69.23 ± 12.43	2.035	0.165	0.28
CD8 + CD45RA + CD95+	44.70 ± 16.73	52.31 ± 13.48	3.495	**0.036**	**–0.50**
CD8 + CD45RO + CD95+	38.68 ± 16.79	27.91 ± 11.76	8.577	**0.017**	**0.71**
CD4 + /CD8+	1.70 ± 0.85	2.00 ± 1.11	0.818	0.369	-0.30

The percentages of naïve T cell subsets (CD4 + CD45RA + , CD8 + CD45RA + , CD4 + CD45RA + CD95 + , and CD8 + CD45RA + CD95 + T cell subsets) were lower, and the percentages of memory T cell subsets (CD4 + CD45RO + , CD4 + CD95 + , CD4 + CD45RO + CD95 + , CD8 + CD45RO + , CD8 + CD45RO + CD95 + T cell subsets) were higher in patients with SCZ than in HCs. The *p*_*FDR*_ was adjusted *p* value corrected for FDR correction.

*SCZ* schizophrenia, *HC* healthy control, *FDR* false discovery rate correction.

**Table 4:** Intracellular cytokine levels in CD8 + CD45RA + /CD45RO + CD95 + T cell subsets.

%	SCZ	HC	*F*	*p*_*FDR*_	*Cohen’s d*
CD8 + CD45RA + CD95+					
IL-1β	9.25 ± 4.88	7.04 ± 4.24	6.662	**0.048**	**0.48**
IL-6	28.55 ± 17.65	21.34 ± 13.94	3.104	0.104	0.45
TNF-α	8.04 ± 6.97	6.24 ± 4.84	2.813	0.131	0.30
INF-γ	3.21 ± 2.37	3.41 ± 3.10	0.964	0.387	-0.07
CD8 + CD45RO + CD95+					
IL-1β	9.58 ± 4.64	7.18 ± 4.28	7.905	**0.022**	**0.54**
IL-6	37.20 ± 20.47	28.34 ± 17.31	6.823	**0.022**	**0.47**
TNF-α	18.85 ± 10.36	14.80 ± 8.79	5.203	**0.035**	**0.42**
INF-γ	9.08 ± 4.87	8.63 ± 5.76	1.320	0.274	0.08

The level of IL-1β derived from CD8 + CD45RA + CD95+ and CD8 + CD45RO + CD95 + T cell subsets and the levels of IL-6, TNF-α derived from CD8 + CD45RO + CD95 + T cell subsets were higher in patients with SCZ than in HCs. The *p*_*FDR*_ was adjusted *p* value corrected for FDR correction.

*SCZ* schizophrenia, *HC* healthy control, *FDR* false discovery rate correction.

### Subcortical volume and cortical thickness

Regarding subcortical volumes, the volumes of the ventricles, left and right CPs were larger in the SCZ group than in the HC group. The average thicknesses of the left and right cerebral hemisphere were thinner in the SCZ group than in the HC group (Table 5). Although the differences were not significant between the groups, comparisons of left and right IPL thickness were presented in Table 5, as IPL_R was the mediator in the following mediation and moderated mediation models. Other subcortical volumes and cortical thickness measures that demonstrated statistically significant group differences showed non-significant correlations with immunosenescence markers or MCCB scores or failed to mediate the effects of immune dysregulation on cognitive deficits.

**Table 5:** Comparison of subcortical volumes and cortical thicknesses between groups.

	SCZ	HC	*F*	*p*_*FDR*_	*Cohen’s d*
ICV (cm^3^)	1575.12 ± 19.94	1585.10 ± 22.89	1.235	0.270	–0.46
*Subcortical volume (cm*^*3*^*)*					
Lateral ventricle _L	13.90 ± 7.24	8.19 ± 3.60	11.469	**1.20** **×** **10**^**–5**^	**0.99**
Lateral ventricle _R	11.81 ± 6.27	7.45 ± 3.29	10.475	**1.80** **×** **10**^**–5**^	**0.87**
3^rd^ ventricle	1.83 ± 0.76	1.31 ± 0.49	9.068	**4.35** **×** **10**^**–5**^	**0.81**
4^th^ ventricle	2.06 ± 0.58	1.78 ± 0.51	7.337	**2.39** **×** **10**^**–4**^	**0.51**
CP_L	0.60 ± 0.19	0.56 ± 0.17	4.853	**0.004**	**0.22**
CP_R	0.64 ± 0.20	0.58 ± 0.21	9.102	**4.35** **×** **10**^**–5**^	**0.29**
*Cortical thickness (mm)*					
Thickness _L	2.45 ± 0.09	2.52 ± 0.07	7.525	**0.002**	**–0.87**
Thickness _R	2.44 ± 0.09	2.50 ± 0.07	6.683	**0.004**	**–0.74**
IPL_L	2.41 ± 0.16	2.46 ± 0.13	3.042	0.132	–0.34
IPL_R	2.33 ± 0.23	2.36 ± 0.21	0.631	0.652	–0.14

The volumes of the ventricles, left and right CPs were larger in patients with SCZ than in HCs. The average thickness of the left and right cerebral hemispheres was thinner in patients with SCZ than in HCs. The thickness of left or right IPL was not significantly different between the groups. The *p*_*FDR*_ was adjusted *p* value corrected for FDR correction.

*ICV* total intracranial volume, *CP* choroid plexus, *IPL* inferior parietal lobule, *SCZ* schizophrenia, *HC* healthy control, *FDR* false discovery rate correction.

The sensitivity analyses demonstrated that the key findings in comparisons of immunosenescence markers, structural imaging data, and cognitive manifestations remained statistically significant even when nonparametric Mann–Whitney U test were applied, indicating the robustness of the results (Supplementary Materials).

### Immunosenescence markers, structural imaging data, and cognitive manifestations

**Fig. 2:**
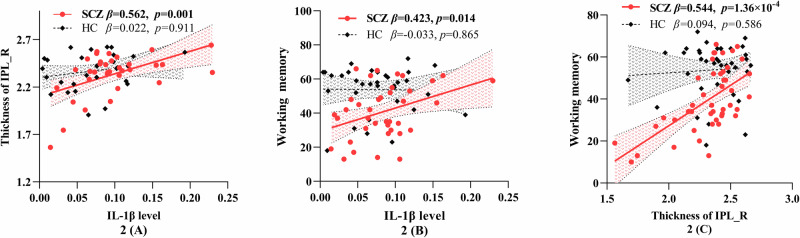
Correlations in different groups. **A**–**C** The level of IL-1β derived from CD8 + CD45RA + CD95 + T cell subsets was positively correlated with the IPL_R thickness and working memory, and the IPL_R thickness was also positively correlated with working memory in patients with SCZ, but those correlations were not significant in HCs. IPL inferior parietal lobule, SCZ schizophrenia, HC healthy control.

**Fig. 3:**
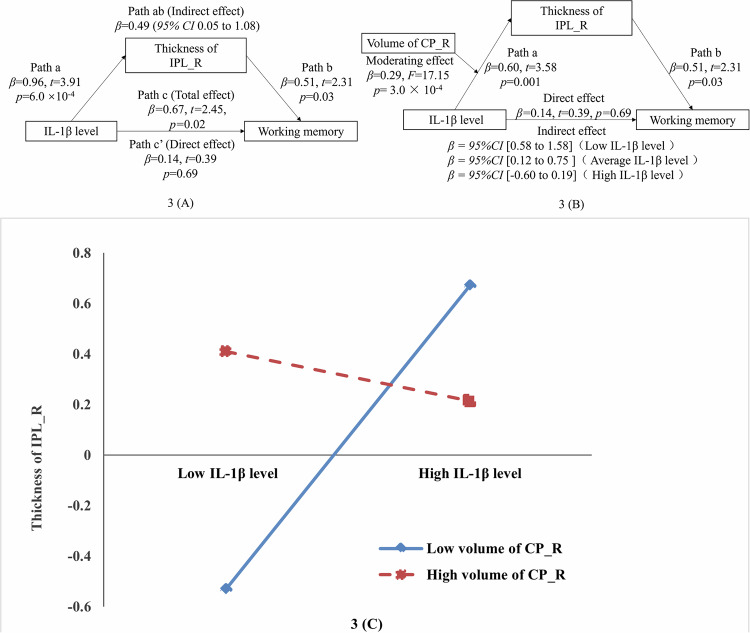
Mediation models in SCZ. **A** Mediation analysis: X: The level of IL-1β derived from CD8 + CD45RA + CD95 + T cells; Mediator: IPL_R thickness; Y: Working memory. **B** Moderated mediation analysis: X represents the level of IL-1β derived from CD8 + CD45RA + CD95 + T cells; Mediator: IPL_R thickness; moderator: CP_R volume; Y: Working memory. **C** Moderation of effect of the above IL-1β level on IPL_R thickness by CP_R volume. IPL inferior parietal lobule, CP choroid plexus.

In the preliminary analyses, we systematically examined correlations among all cognitive metrics (seven subscale scores and one total score of MCCB), immunosenescence markers (T cell subpopulation percentages and intracellular cytokine levels), and structural imaging data (subcortical volumes and cortical thicknesses). The results demonstrated that intracellular IL-1β level (derived from CD8 + CD45RA + CD95 + T cells) exhibited significant pairwise correlations with working memory and cortical thickness in the bilateral IPL (IPL_L and IPL_R) and bilateral supramarginal gyri (SMG_L and SMG_R) in the SCZ group. However, these correlations were not significant in the HC group. The sensitivity analyses demonstrated that the findings remained statistically significant when Spearman correlation analyses were employed (Supplementary Materials). In subsequent mediation analyses, with the level of IL-1β (derived from CD8 + CD45RA + CD95 + T cell subpopulations) as the independent variable, working memory as the dependent variable, IPL_L, IPL_R, SMG_L and SMG_R thickness as the mediator respectively, with sex, age, ICV and hCPR level as covariates, only the IPL_R thickness emerged as a statistically significant mediator. The pairwise correlations between the level of IL-1β, IPL_R thickness, and working memory were shown in Fig. 2A–C, the corresponding mediation model was shown in Fig. 3A, in which the indirect and total effects were significant, whereas the direct effect was not. The mediation models with IPL_L, SMG_L, and SMG_R thickness as the mediator, respectively, were detailed in the supplementary materials. Further, in the moderated mediation analysis, the right CP volume was a moderator in the relationship between the above IL-1β level and the IPL_R thickness (Fig. 3B). Specifically, the IPL_R thickness mediated the association between the above IL-1β level and working memory at low (16th percentile) and moderate (50th percentile) levels of CP_R volume, but not at high level of CP_R volume (84th percentile) (Fig. 3B), which suggested that the volume of CP_R negatively moderated the positive association between the IL-1β level and the IPL_R thickness (Fig. 3C). The mediating and moderated mediating effects were not significant in the HCs.

### Effect of antipsychotics

The potential effects of antipsychotic medications were rigorously considered in this study. Specifically, analyses of CPZ revealed no significant correlations with immunosenescence, cognitive or imaging measurements (Supplementary Materials, all *ps* å 0.05). Second, the effects of antipsychotic types were further examined. The T cell phenotype, cytokine level, neurocognition, subcortical volumes, and cortical thicknesses between patients treated with and without clozapine /olanzapine were compared with no statistically significant differences between groups. Only the percentage of senescent CD8 + CD45RO + CD95 + T cells show a possible trend toward a statistically significant higher level in those with clozapine than those without (Supplemental Materials). The sample sizes using other antipsychotics, mood stabilizers, and antidepressants were too small to conduct statistically significant comparisons. Details on the number of patients administered for each psychopharmacological agent and the corresponding dosage were provided in the supplementary materials.

## Discussion

The results suggest that immunosenescence-related T cell phenotypes are more pronounced in SCZ patients than in HCs and are associated with memory deficit. The IPL_R thickness mediated the association between the immunosenescence makers and working memory, and most importantly, this mediating effect was itself moderated by the CP_R volume.

The present findings support the concept of senescent remodeling of the immune system in SCZ^31^, as evidenced by the presence of naïve and memory T cell subsets labeled with combined CD45 and CD95 markers, which manifested SASP. We found that the percentages of naïve T cell subsets were lower and the percentages of memory T cell subsets were higher in the SCZ group than in the HC group. Moreover, the cytokine secretion functions of naïve and memory T cells were altered in the SCZ group compared with the HC group. These findings are consistent with those reported for aging and other neurological diseases^32^. The differentiation of T cells toward memory phenotypes, coupled with suppressed thymic output of naïve T cells, may indicate diminished immune system regenerative capacity and compromised responses to novel pathogens^4^. The elevated CD95 + T cell subsets further suggest increased apoptosis susceptibility, which may disrupt T cell homeostatic balance through enhanced pro-apoptotic signaling^33,34^ Mechanistically, the SASP profile in T cells may perpetuate neuroinflammation through cross-talk with glia, potentially amplifying neuroinflammatory cascades and accelerating neuronal aging^35^. These pathological processes may critically underlie the cognitive deficits in SCZ patients^36^. Notably, some earlier studies revealed a higher naïve T cell count in patients with SCZ than in HCs. This was probably because the average age in earlier studies was younger^11^, which may impact the T cell composition, given that the functionality and distribution of T cell subpopulations vary with age and disease stage^5^. Further research targeting the measurement of these T cell markers in the context of various disease stages and across different age ranges is warranted.

**Fig. 4:**
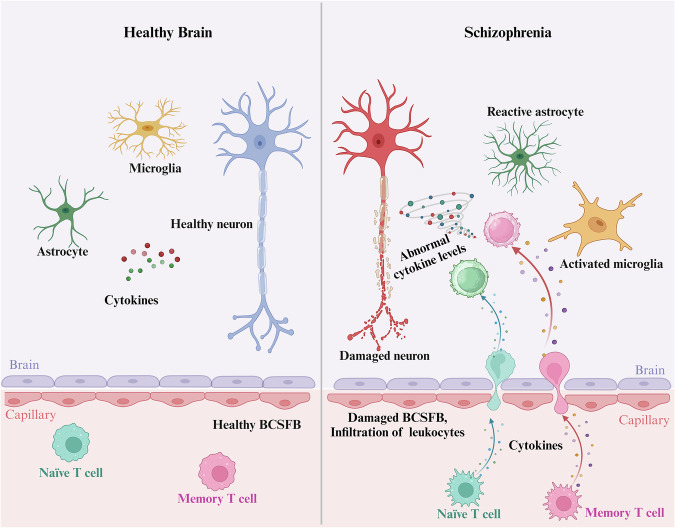
Illustration of bidirectional communication between the CNS and immune system. The illustration of bidirectional communication between the CNS and immune system in the healthy brain and SCZ. In SCZ, peripheral immune cells could penetrate the damaged BCSFB (in which cytokines may be key factors) and induce neuronal dysfunction during the process of immunosenescence. BCSFB blood-cerebrospinal fluid barrier, CNS central nervous system, SCZ schizophrenia.

In this study, the level of IL-1β derived from CD8 + CD45RA + CD95+ naïve T cell subsets was higher in patients with SCZ than HCs and was positively associated with working memory in SCZ. Both rodent and human studies have suggested that age-related downregulation of naïve markers and upregulation of memory markers in CD4+ and CD8 + T cells contribute to impaired cognition^37,38^. Moreover, numerical alteration of T cell subsets may impair their functionality^32^. For example, in vitro studies, naïve T cells generated from human and mouse models of aging exhibit decreased proliferative activity and responsiveness to TCR activation, as well as altered production of cytokines^39^. Based on these findings, we speculated that the increased intracellular IL-1β levels could reflect a compensatory process for the reduced number of CD8 + CD45RA + CD95+ naïve T cells. We also found that the above IL-1β level was positively associated with working memory in SCZ. Cytokines are multifunctional mediators that can promote or inhibit inflammatory processes in a context-dependent manner^32^. Generally, the role of IL-1β is regarded as detrimental in the context of neurodegeneration^40^, however, an increasing body of clinical and experimental evidence has validated its beneficial properties in neuronal homeostasis and cognitive performance by maintaining the equilibrium of neurodegeneration and neuroprotection. Indeed, consistent with our findings, IL-1 appeared beneficial to cognitive function by providing trophic support to neurons and neurogenesis^41^. In animal models, hippocampus IL-1β levels were considered to change in an age-dependent manner, with at least a temporarily beneficial effect on learning and memory^42^. Elevated IL-1β in naïve T cells could also reflect compensatory mechanisms to counteract neuronal hypoactivity by transiently boosting synaptic transmission. Preclinical studies show that low dose IL-1β enhances hippocampal neurogenesis and spatial memory^43^, but chronic exposure may drive excitotoxic damage through sustained N-methyl-D-aspartate receptor activation over time^44^. On the other hand, it is plausible that cognitive functions might downregulate peripheral inflammation via brain-to-immune signaling, such as vagus nerve mediated cholinergic anti-inflammatory pathways^45^ or HPA axis suppression of pro-inflammatory cytokines, indirectly shaping T cell phenotypes^46^. Longitudinal studies tracking IL-1β dynamics and cognitive trajectories are needed to disentangle causality. The reason behind the IL-1β level in the memory T cell subsets not being related to working memory in this study is not immediately clear. The potentially distinct functions of cytokines in naïve and memory T cells need to be addressed to elucidate the process of T cell progression toward exhaustion or senescence. Many other cytokines, e.g., TNF-α, IL-6, and IFN-γ, are the hallmarks of immunosenescence^8,9^. However, in the current study, the levels of these cytokines were not found to be associated with structural imaging or cognitive measurements. This discrepancy should be clarified in future work. In this study, IPL_R thickness mediated the effect of intracellular level of IL-1β on working memory scores. Furthermore, the volume of CP_R moderated the association between the IL-1β level and IPL_R thickness. The interplay between the CNS and immune system during aging is characterized by bidirectional dependency^32^, in which the CP is of pivotal importance. The CP, the principal structure of the blood-CSF barrier, lines all ventricles and serves as the barrier that regulates immune-to-brain communication, where cytokines might be the key molecules^32^. For example, IL-1β had effects on the gateway function of the CP by inhibiting trans-epithelial ion flux and changes in permeability^47^. Bidirectional communication between the CNS and immune system can contribute to body protection when properly orchestrated^32^. The CP enlargement is a well-established pathological feature in SCZ, observed even in first-episode, antipsychotic-naïve patients^21^. Our findings reveal that IPL_R thickness mediates the association between IL-1β and working memory only at low-to-moderate, but not high CP_R volumes. At high level of CP volume, structural deterioration (e.g., fat deposition, epithelial hypersecretion, and cystic formations) may disrupt the integrity of blood-cerebrospinal fluid barrier (BCSFB)^19,21^, thus decoupling peripheral IL-1β levels from their effects on IPL_R and cognitive function. This is supported by recent evidence linking CP enlargement to BCSFB dysfunction, where excessive expansion possibly impairs molecular exchange and neuroimmune signaling^48^ (Fig. 4). While both the IPL_R and IPL_L showed baseline correlations with IL-1β levels and working memory performance, only the right hemisphere exhibited statistically significant mediation and moderated mediation effects. This likely reflects the intrinsic hemispheric lateralization in SCZ. First, previous studies reported neuroinflammatory lateralization, such as greater microglial density in the right versus left hemispheres in SCZ^49^, potentially amplifying the effects of IL-1β on IPL_R plasticity. Furthermore, previous evidence indicated that CP_R volume was significantly larger than CP_L in SCZ patients^22^, possibly suggesting heightened right-sided CP activity in mediating the immune-CNS crosstalk. The bilateral IPL supports working memory, as shown in functional MRI and electroencephalogram (EEG) studies^23,50^. For example, the IPL is consistently activated during n-back working memory tasks, and older vs. younger individuals exhibit decreased activation of the IPL^51^. The theta coupling (in EEG) between the parietal and prefrontal cortices has been shown to predict individual working memory capacity, and the frontoparietal phase coupling induced by transcranial alternating current stimulation leads to improved working memory, whereas desynchronization impairs it^23^. Further, working memory deficit is also related to abnormal IPL activation in SCZ^24^. In accordance with our findings, the IPL may contribute to the association between peripheral inflammatory signals and CNS changes in SCZ. Gray matter volume reductions in the IPL were found in multiple inflammatory subgroups of patients with SCZ compared to HCs^52^.

The effect of psychopharmacological agents on immunosenescence was assessed in this study. While certain biomarkers of premature aging are associated with antipsychotics in SCZ^53^, no consensus exists regarding immunosenescence-related T cell phenotypes. Crucially, CPZ showed no correlation with immunosenescence markers in this study. Given known immunomodulatory properties of clozapine^54^, we specifically compared immunosenescence markers between clozapine-exposed and non-exposed patients. No statistically significant differences emerged, though a trend toward higher senescent CD8⁺CD45RO⁺CD95⁺ T cells was observed in the clozapine treated group. This contrasts with previous suggestions that anti-inflammatory effects of clozapine might delay immunosenescence^55^. Potential reasons for inconsistent findings include limited sample sizes, population heterogeneity, disease severity, and illness duration. Notably, alterations in T cell subsets were observed even in antipsychotic-naïve first-episode SCZ^56^, making it challenging to disentangle medication effects from disease progression in chronic patients with complex treatment histories^53^. Similarly, olanzapine exposure showed no significant impact on immunosenescence markers. Analyses for other agents were precluded by insufficient sample sizes (antipsychotics, mood stabilizers, antidepressants; *n* = 1–9). The effects of psychopharmacological agents on T cell senescence and cytokine regulation in SCZ remain poorly understood. Systematic clinical and biological assessments are needed to elucidate the interplay between antipsychotics, immunosenescence, and SCZ. No significant association was observed between exposure to antipsychotics (CPZ, clozapine, or olanzapine) and cognitive performance. This aligns with the literature where evidence remains inconsistent: while some studies report detrimental effects^57^, others show neutral or even beneficial outcomes^58^. Developing improved therapies for cognitive deficits requires a deep understanding of SCZ-specific pathophysiology. Our findings on immunosenescence may support exploring novel immunomodulatory treatment approaches clinically.

### Limitation

First, the cross-sectional design limits the causal interpretations of immunosenescence-cognition relationships. Future longitudinal detections of immunosenescence markers in both ultra-high-risk and first-episode SCZ cohorts are warranted. Second, the current analyses were restricted to structural brain imaging data. Future studies should integrate multimodal imaging biomarkers to provide a more comprehensive characterization of neuro-immune interactions. Importantly, the absence of significant associations between structural abnormalities in prefrontal, parietal, and other cognition-relevant regions with immune-cognitive measures reinforces the necessity for larger longitudinal cohorts to explore potential mechanistic links. Third, although we observed no significant associations between antipsychotic exposure (CPZ, clozapine, or olanzapine), immunosenescence markers, and cognitive scores, the potential impact of chronic antipsychotic treatment cannot be excluded. Even considering the established immunomodulatory effects of psychopharmacological agents^59–61^, limited sample size precluded robust subgroup analyses for other antipsychotics, mood stabilizers, and antidepressants in current study. Comprehensive evaluations of these agents in first-episode, treatment-naïve cohorts are warranted to delineate their specific neurobiological and clinical effects. Fourth, we only measured a limited panel of cytokines within specific T cell subsets. Future investigations employing comprehensive SASP profiling will be crucial to fully elucidate the immunosenescence-aging phenotype in SCZ.

## Conclusions

The present study indicated that IPL_R and CP had a significant moderated mediation effect on the impact of immunosenescence-related T cell phenotypes on cognitive deficit in schizophrenia patients. Reversing immunosenescence can prevent chronic inflammation and neurodegeneration^32^, and therapeutic approaches targeting the immune environment of the CP can offer protective benefits for the brain. Thus, these results indicate that immunosenescence and the CP could serve as promising targets for procognitive treatments in schizophrenia^62^. Further research is needed to fully understand the underlying mechanisms and to determine the potential of immune-based interventions for this population.

## Supplementary information

Immunosenescence-related T cell phenotypes, structural brain imaging, and cognitive impairment in patients with schizophrenia: A moderated mediation analysis
