# Supplementary material for: Immunosenescence-related T cell phenotypes, structural brain imaging, and cognitive impairment in patients with schizophrenia: a moderated mediation analysis
Source: Schizophrenia (Heidelb). 2025 Jul 18;11(1):101. doi: 10.1038/s41537-025-00650-w (PMC12274616; doi:10.1038/s41537-025-00650-w)
Supplement: Supplementary file 1 — Immunosenescence-related T cell phenotypes, structural brain imaging, and cognitive impairment in patients with schizophrenia: A moderated mediation analysis [file 41537_2025_650_MOESM1_ESM.docx]

**Immunosenescence-related T cell phenotypes, structural brain imaging, and cognitive impairment in patients with schizophrenia: A moderated mediation analysis**

***Supplementary Material***

***Method***

***Immunophenotyping***

***Measurements of the percentages of naïve and memory T cell subsets and the levels of intracellular cytokines***

After 10 hours overnight fasting, 5ml of whole blood was collected in heparin lithium-anticoagulant tube during 6:00 a.m.-7:00 a.m. and immediately processed within half an hour. The flow cytometry was used to count the percentages of specific lymphocyte subsets, including CD4+, CD4+CD45RA+, CD4+CD45RO+, CD4+CD95+, CD4+CD45RA+CD95+, and CD4+CD45RO+CD95+ T cell subsets; and CD8+, CD8+CD45RA+, CD8+CD45RO+, CD8+CD95+, CD8+CD45RA+CD95+, and CD8+CD45RO+CD95+ T cell subsets. We also detected the levels of intracellular cytokines in CD8+CD45RA+CD95+ and CD8+CD45RO+CD95+ T cell subsets, including IL-1β, TNF-α, IL-6 and IFN-γ.

For the percentage of T cell subsets, the procedures of surface staining were as following, (1) 100μl of whole blood samples were incubated with 1μl each of CD3, CD4, CD8, CD45RO/CD45RA and CD95 antibodies for 15 minutes at room temperature (RT) in dark. (2) 2ml of lysis buffer was added to lyse red blood cells for additional 5 minutes in dark. (3) The samples were subsequently centrifuged at 350g for 5 minutes and the supernatants were discarded. (4) Cell pellets were washed with 1ml cell staining buffer, centrifuged at 350g for 5 minutes and resuspended in 100μl cell staining buffer, which was immediately acquisited by a flow cytometer (Beckman Coulter, CytoFLEX S).

For the detection of the level of intracellular cytokines, (1) Each 200ul whole blood samples were added with 2ml of lysis buffer to lyse red blood cells for 5 minutes in dark, subsequently centrifuged at 350g for 5 minutes, and discarded supernatant. (2) Cell pellets were washed with 1ml cell staining buffer, centrifuged at 350g for 5 minutes and resuspended in 65μl cell staining buffer and stained with each of 1μl anti-human CD3, CD4, CD8, CD95, CD45RO/CD45RA antibody for 15 minutes at RT in dark. (3) Cell pellets were washed with 1ml cell staining buffer, centrifuged at 350g for 5 minutes and resuspended in 65μl cell staining buffer. (4) The cells were stained with the fixable viability marker (Zombie-NIR) and added with 150μl fix/perm for fixation and permeabilization for 20 minutes at RT in dark, then were washed with 800ul perm/wash buffer and centrifuged at 350g for 5 minutes twice, prior to intracellular staining. (5) Cell pellets were then resuspended in 50μl perm/wash buffer and stained with 2μl each of anti-human TNF-α, IL-1β, IL-6 and INF-γ antibodies for 20 minutes at RT in dark. (6) Cell pellets were washed with 1ml cell staining buffer, centrifuged at 350g for 5 minutes and resuspended in 100μl cell staining buffer, which was immediately acquisited by a flow cytometer (Beckman Coulter, CytoFLEX S). (See Supplemental Table 1 for key reagent sources).

We collected a total of 5000 events for each sample and data were analyzed using CytExpert. The percentage of T cell subsets and the level of cytokines were presented. The gating strategy is illustrated in Supplemental Figure 1.

Supplemental Table 1. Key resources table

| Reagent or Resource | Identifier | Source |
| --- | --- | --- |
| PE/Cyanine7 anti-human CD3 | UCHT1; Cat#300419 | Biolegend |
| FITC anti-human CD4 | RPA-T4; Cat#300505 | Biolegend |
| PerCP/Cyanine5.5 anti-human CD8 | SK1; Cat#344709 | Biolegend |
| APC anti-human CD95 (Fas) | DX2; Cat# 305611 | Biolegend |
| Brilliant Violet 510™ anti-human CD45RO | UCHL1; Cat# 304245 | Biolegend |
| Brilliant Violet 605™ anti-human CD45RA | UCHL1; Cat# 304245 | Biolegend |
| RBC Lysis Buffer (10X) | Cat#420301 | Biolegend |
| Cell Staining Buffer | Cat# 420201 | Biolegend |
| Zombie NIR™ Fixable Viability Kit | Cat#423105 | Biolegend |
| Cyto-Fast™ Fix/Perm Buffer Set | Cat#426803 | Biolegend |
| Perm/Wash Buffer | Cat#421002 | Biolegend |
| Brilliant Violet 421™ anti-human TNF-αAntibody | MAb11; Cat# 502931 | Biolegend |
| Brilliant Violet 650™ anti-human IFN-γ Antibody | 4S.B3; Cat# 502538 | Biolegend |
| PE anti-human IL-6 Antibody | MQ2-13A5;Cat# 501107 | Biolegend |
| Alexa Fluor® 647 anti-human IL-1β Antibody | JK1B-1; Cat# 508208 | Biolegend |

Supplemental Figure 1. Gating strategy by flow cytometry.

| CD3+  (1) | CD4+  (2) | CD4+  (3) | CD4+    (4) |
| --- | --- | --- | --- |
| CD8+    (5) | CD8+    (6) | CD8+    (7) | CD8+CD45RA+CD95+    (8) |

Representative scatterplots were shown to illustrate the gating strategy of T cell subsets and intracullar cytokines. (1) CD4+ and CD8+ T cells. (2) CD4+CD45RA+/RO+ T cells; (3) CD4+CD95+ T cells; (4) CD4+CD45RA+/RO+CD95+ T cells. (5) CD8+CD45RA+/RO+ T cells; (6) CD8+CD95+ T cells; (7) CD8+CD45RA+/RO+CD95+ T cells. (8) The intracellular level of IL-1β in CD8+CD45RA+CD95+ T cells. The detecion of the level of other intracellular cytokines in the CD8+CD45RA+CD95+ and CD8+CD45RO+CD95+T cells were almost the same.

***Structural MRI data acquisition and preprocessing***

The T1-weighted high-resolution structural brain imaging data were acquired using a Siemens Prisma 3.0 T MRI scanner equipped with a 64-channel head coil.

*MRI scanning:* the participants were reminded to avoid moving, falling asleep, or opening their eyes during the scanning procedure. The participants wore earplugs to reduce machine noise. And spongy pads were placed to restrict head movement.

*Scanner parameters:* A magnetization-prepared rapid acquisition gradient echo sequence was used to collect data with the following parameters: repetition time (TR) =2,530 ms, echo time (TE) =2.98 ms, field-of-view (FOV) =256×224 mm, flip angle (FA) =7°, matrix size =256×224, thickness/gap =1/0 mm, and inversion time (TI) =1100 ms.

*Operational details:* the FreeSurfer v6.0 (<https://surfer.nmr.mgh.harvard.edu/>) was used to conduct the automated and validated segmentation of subcortical volume and cortical thickness: including motion correction, non-brain tissue removal, surface smoothing, image reconstruction, and segmentation. All images were manually checked for quality control. Ventricle and choroid plexus (CP) volumes, seven regional subcortical volumes per hemisphere (nucleus accumbens, amygdala, caudate, hippocampus, pallidum, putamen, and thalamus), 34 regional cortical thicknesses per hemisphere and total intracranial volume were extracted for statistical analyses. The cortical parcellations were based on the Desikan-Killiany atlas.

***Results***

***1. Sensitivity Analyses***

The Shapiro-Wilk test was used to confirm distributions for all variables. We further conducted between-group comparisons using the Mann-Whitney U test and analyzed interrelationships between variables using Spearman's rank-order correlation for the following non-normal distributed data.

(1) We conducted the Mann-Whitney U test for non-normal distributed data; results were shown in Table 1.

Table 1. Mann-Whitney U test for non-normal distributed data

|  | SCZ  Median (25–75%) | HC  Median (25–75%) | *U* | *p* |
| --- | --- | --- | --- | --- |
| Attention/vigilance | 30.50(27.00-39.50) | 47.00(41.00-56.00) | -5.125 | **2.98×10^-7^** |
| Verbal learning | 45.00(33.00-57.00) | 56.00(49.00-61.00) | -3.211 | **0.001** |
| Working memory | 35.00(27.50-49.00) | 58.00(49.00-63.00) | -5.004 | **5.61×10^-7^** |
| CD4+CD45RA+CD95+ | 23.25(19.58-26.77) | 27.98(19.98-33.32) | -2.241 | **0.025** |
| CD8+ | 34.58(29.16-47.67) | 33.01(27.82-38.46) | -0.995 | 0.320 |
| TNF-α(CD8+CD45RA+CD95+) | 6.16(2.42-10.68) | 6.55(1.67-10.21) | -0.580 | 0.562 |
| INF-γ(CD8+CD45RO+CD95+) | 9.53(5.60-11.75) | 6.73(5.01-11.94) | -0.898 | 0.369 |
| Lateral ventricle _L | 11.84(9.88-15.52) | 7.45(5.83-9.83) | -4.613 | **4.00×10^-6^** |
| Lateral ventricle _R | 10.63(7.80-14.26) | 6.37(5.14-9.40) | -4.159 | **3.20×10^-5^** |
| 4th ventricle | 1.99(1.75-2.33) | 1.66(1.50-1.91) | -2.970 | **0.003** |
| CP_R | 0.67(0.50-0.75) | 0.54(0.45-0.63) | -2.020 | **0.043** |
| IPL_L | 2.42(2.36-2.53) | 2.46(2.38-2.58) | -1.395 | 0.163 |
| IPL_R | 2.38(2.24-2.47) | 2.39(2.25-2.53) | -0.381 | 0.703 |

Results from Mann-Whitney U test for the above non-normal distributed data were consistent with those from analysis of covariance.

(2) We conducted Spearman's rank-order correlation, the results showed that the level of IL-1β derived from CD8+CD45RA+CD95+ T cell subpopulations was positively correlated with the IPL_R thickness (*r*=0.414, *p*=0.017) and working memory(*r*=0.415, *p*=0.013), and the IPL_R thickness was also positively correlated with working memory (*r*=0.538, *p*=1.17×10^-4^); these correlations were not significant in HCs (*r*=0.272, *p*=0.139; *r*=-0.109, *p*=0.560; *r*=-0.009, *p*=0.955, respectively). Results from parametric and non-parametric analyses were consistent.

(3) A comparison of results from parametric and nonparametric test revealed consistent findings, as shown in Table 2.

Table 2. Comparisons of parametric and non-parametric test results

| Comparison​ | Parametric analyses  *F*(*p*) | Non- Parametric analyses *U*(*p*) | Consistent  (Yes/No) |
| --- | --- | --- | --- |
| ANCOVA vs Mann-Whitney U |  |  |  |
| Attention/vigilance | 34.121 (**3.476×10^-7^**) | -5.125 (**2.98×10^-7^**) | **Yes** |
| Verbal learning | 9.355 (**0.003**) | -3.211 (**0.001**) | **Yes** |
| Working memory | 28.714 (**1.536×10^-6^**) | -5.004 (**5.61×10^-7^**) | **Yes** |
| CD4+CD45RA+CD95+ | 3.495 (**0.026**) | -2.241 (**0.025**) | **Yes** |
| CD8+ | 1.350 (0.291) | -0.995 (0.320) | **Yes** |
| TNF-α (CD8+CD45RA+CD95+) | 2.813 (0.131) | -0.580 (0.562) | **Yes** |
| INF-γ (CD8+CD45RO+CD95+) | 1.320 (0.274) | -0.898 (0.369) | **Yes** |
| Lateral ventricle _L | 11.469 (**1.20×10^-5^**) | -4.613 (**4.00×10^-6^**) | **Yes** |
| Lateral ventricle _R | 10.475 (**1.80×10^-5^**) | -4.159 (**3.20×10^-5^**) | **Yes** |
| 4th ventricle | 7.337 (**2.39×10^-4^**) | -2.970 (**0.003**) | **Yes** |
| CP_R | 9.102 (**4.35×10^-5^**) | -2.020 (**0.043**) | **Yes** |
| IPL_L | 3.042 (0.132) | -1.395 (0.163) | **Yes** |
| IPL_R | 0.631 (0.652) | -0.381 (0.703) | **Yes** |
| Pearson vs Spearman correlation analysis (SCZ group) |  |  |  |
| IL-1β and IPL_R | 0.562 (0.001) | 0.414 (0.017) | **Yes** |
| IL-1β and working memory | 0.423 (0.014) | 0.415 (0.013) | **Yes** |
| IPL_R and working memory | 0.544 (1.36×10^-4^) | 0.538 (1.17×10^-4^) | **Yes** |

The results from parametric and non-parametric analyses were consistent.

**2. Mediation Models**

Mediation analyses were conducted to evaluate whether cortical thickness mediated the relationship between ​​IL-1β levels​​ and ​​working memory​​. Mediation models (with ​​left and right SMG​​, left IPL as mediators respectively) did not reach statistical significance.


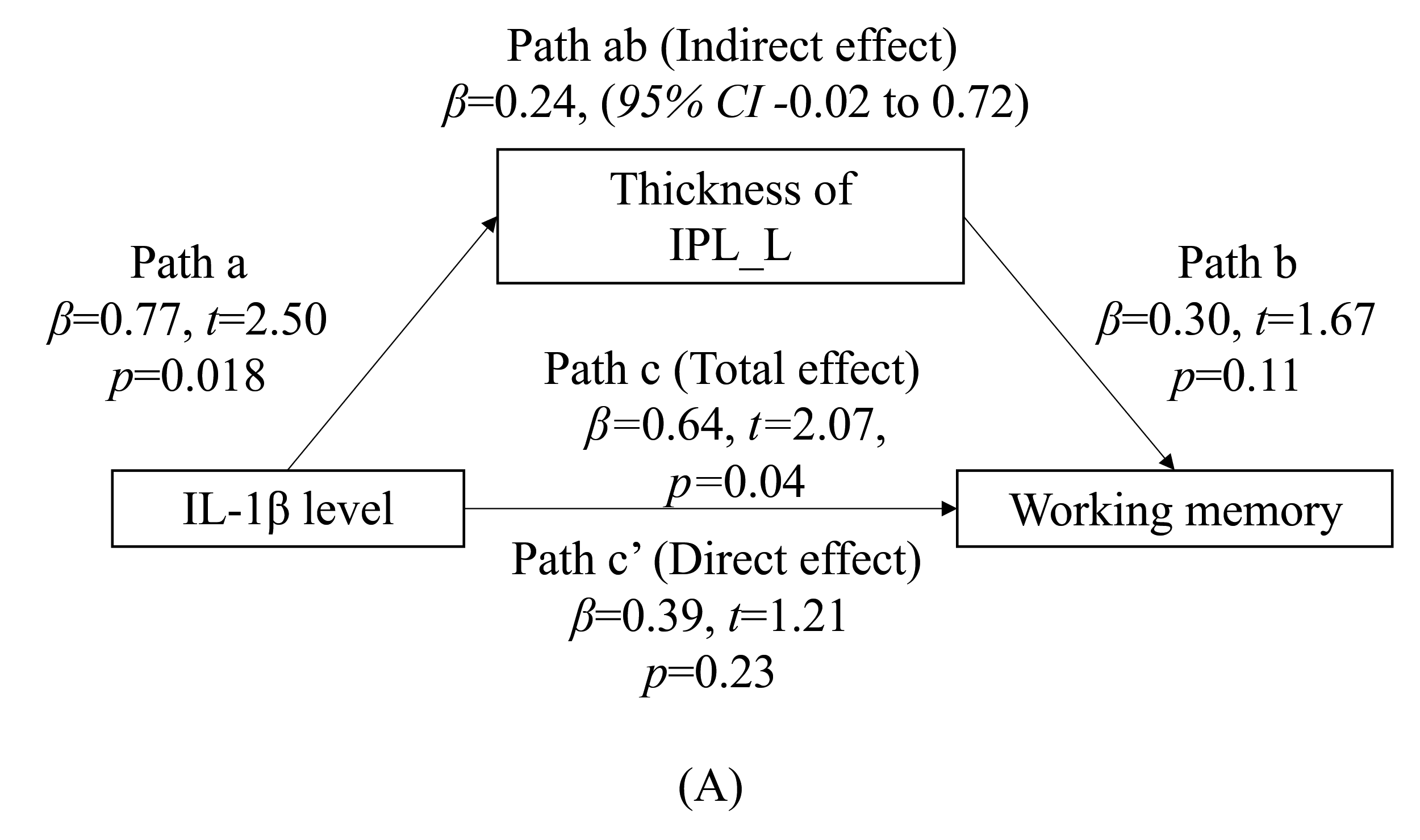

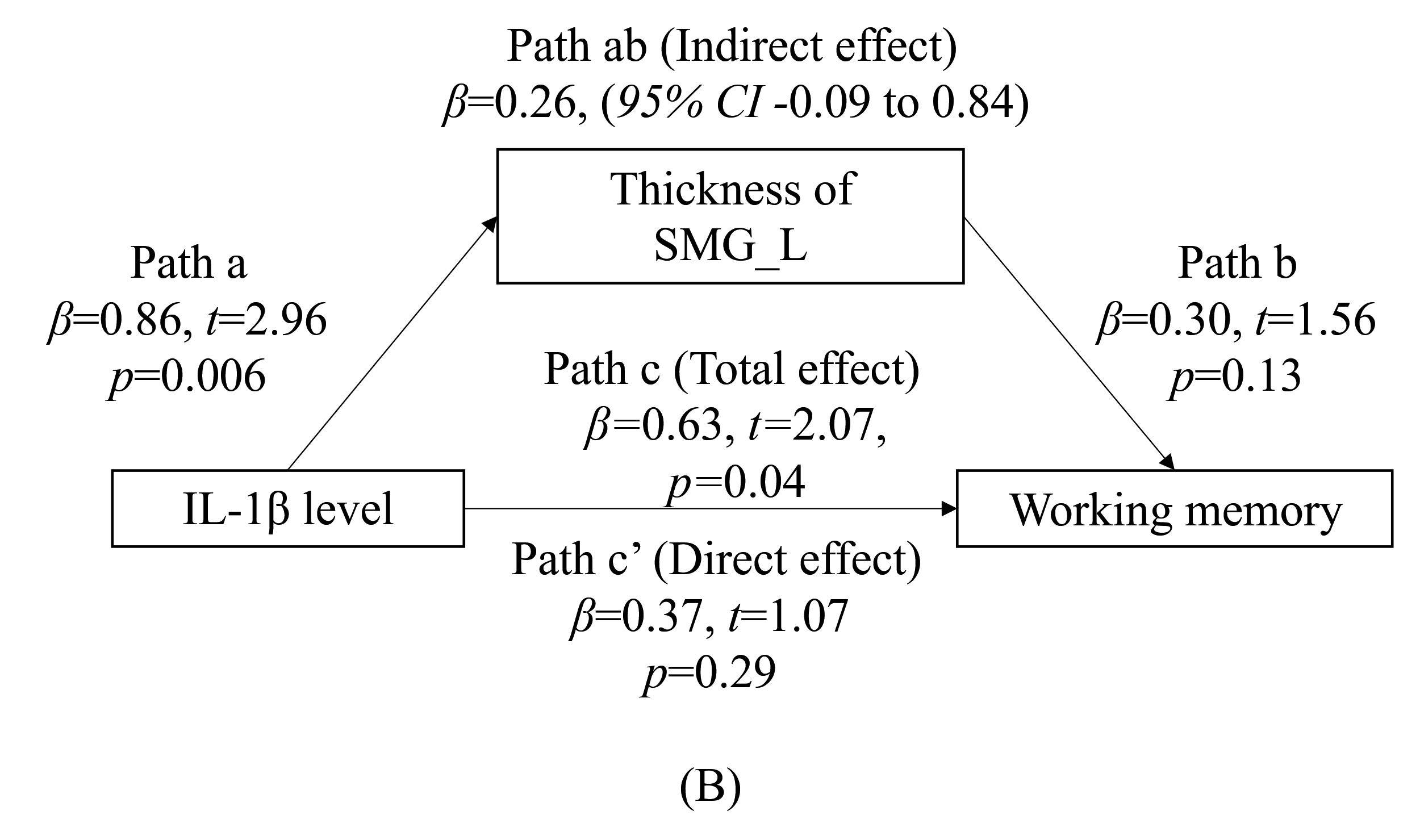

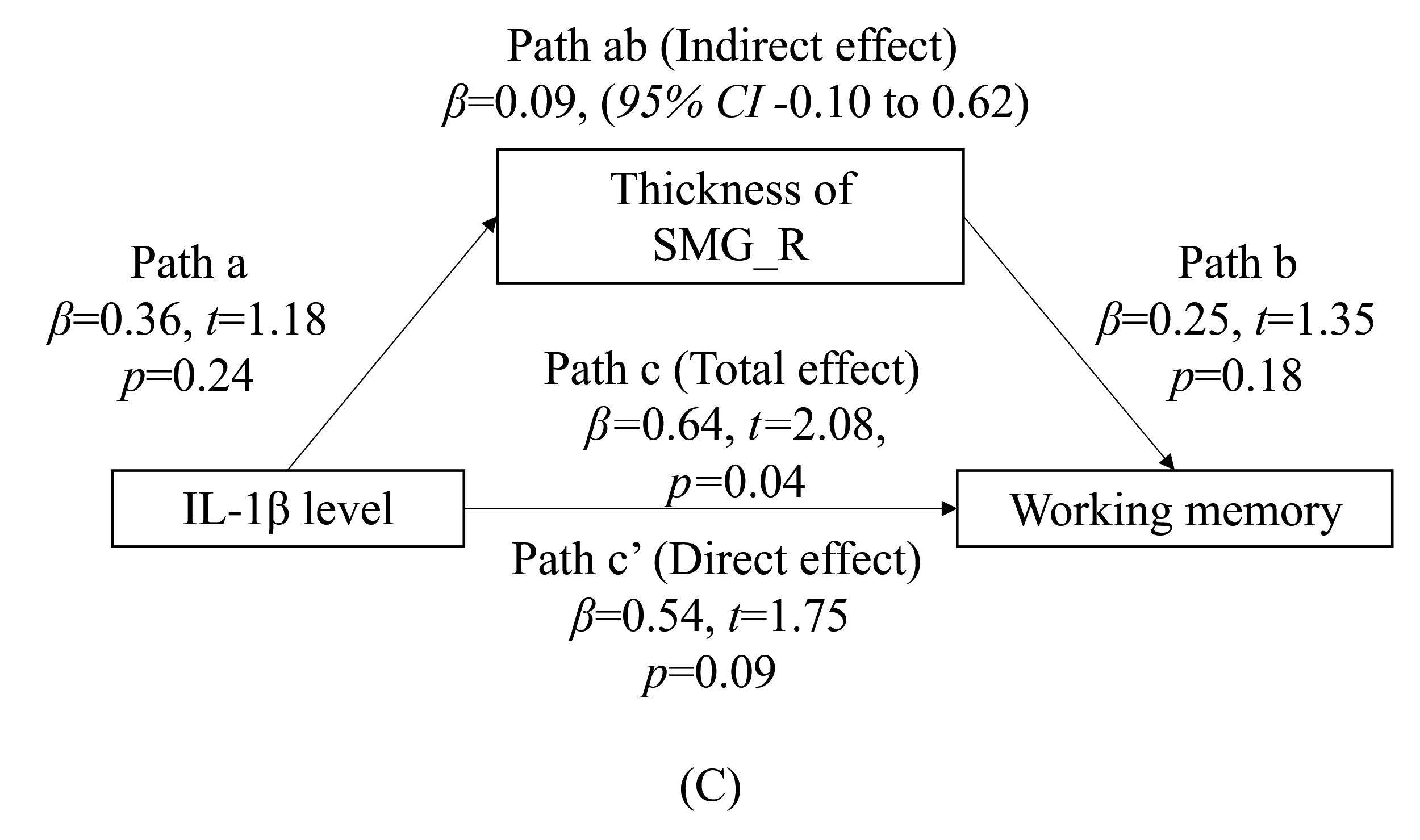


Mediation analysis: X: The level of IL-1β derived from CD8+CD45RA+CD95+ T cells. Mediator: Thickness of (A)IPL_L; (B) SMG_L; and(C) SMG_R. Y: Working memory. The indirect effect and direct effect were not statistically significant in these models.

***3.Effect of antipsychotics***

***(1) The Effect of CPZ***

We performed analyses of the correlation between CPZ and the immunosenescence markers, structural imaging data, and cognitive manifestations using Pearson correlation analysis.

① The CPZ was not statistically correlated with the percentage of CD4+ T cells (*r*=-0.168, *p*=0.327), CD4+CD45RA+ T cells (*r*=0.148, *p*=0.388), CD4+CD45RO+ T cells (*r*=-0.138, *p*=0.368), CD4+CD45RA+CD95+ T cells (*r*=0.240, *p*=0.158), CD4+CD45RO+ CD95+ T cells (*r*=-0.162, *p*=0.346), CD8+ T cells (*r*=-0.003, *p*=0.998), CD8+CD45RA+ T cells (*r*=0.120, *p*=0.486), CD8+CD45RO+ T cells (*r*=-0.117, *p*=0.495), CD8+CD45RA+ CD95+ T cells (*r*=0.111, *p*=0.518) and CD8+CD45RO+ CD95+ T cells (*r*=-0.121, *p*=0.484).

② The CPZ was not statistically correlated with the intracellular level of IL-1β (*r*= -0.127, *p*=0.459), IL-6 (*r*=-0.078, *p*=0.651), TNF-α (*r*=-0.112, *p*=0.516) and INF-γ (*r*=-0.046, *p*=0.789) in naïve CD8+CD45RA+CD95+ T cell subsets and the intracellular level of IL-1β (*r*= -0.144, *p*=0.402), IL-6 (*r*=-0.116, *p*=0.502), TNF-α (*r*=-0.160, *p*=0.350) and INF-γ (*r*=-0.068, *p*=0.695) in memory CD8+CD45RO+CD95+ T cell subsets.

③ The CPZ was not statistically correlated with speed of processing (*r*= -0.034, *p*=0.809), attention/vigilance (*r*= -0.069, *p*=0.632), verbal learning(*r*= 0.024, *p*=0.867), visual learning (*r*= -0.012, *p*=0.933), working memory (*r*= -0.085, *p*=0.548), reasoning problem solving(*r*= -0.247, *p*=0.077), social cognition (*r*= -0.019, *p*=0.896)and total score(*r*= -0.067, *p*=0.640) in MCCB measurement.

④ The CPZ was not statistically correlated with ICV (*r*= -0.048, *p*=0.760);

volume of left lateral ventricle (*r*= -0.124, *p*=0.430), right lateral ventricle (*r*= 0.004, *p*=0.981), 3^rd^ ventricle (*r*= -0.044, *p*=0.774), 4^th^ ventricle (*r*= -0.065, *p*=0.681), CP_L (*r*= -0.063, *p*=0.690),CP_R (*r*= -0.017, *p*=0.912); and the average thickness of left hemisphere(*r*= -0.257, *p*=0.096), right hemisphere (*r*= -0.300, *p*=0.061), the thickness of IPL_L (*r*= -0.133, *p*=0.394) and IPL_R (*r*= -0.139, *p*=0.375).

***(2) The Comparison between Patients with and without Clozapine***

Table 1. MCCB scores

|  | Clozapine  (n=20) | Non_Clozapine  (n=45) | *t* | *p* | *Cohen's d* |
| --- | --- | --- | --- | --- | --- |
| Speed of processing | 45.67±14.54 | 43.10±15.83 | 0.588 | 0.559 | 0.169 |
| Attention/vigilance | 35.11±9.42 | 34.08±11.13 | 0.343 | 0.733 | 0.099 |
| Verbal learning | 41.94±17.44 | 46.56±16.24 | -0.983 | 0.330 | 0.274 |
| Visual learning | 37.22±10.69 | 42.29±12.19 | -1.524 | 0.133 | 0.442 |
| Working memory | 37.11±12.38 | 38.61±16.14 | -0.351 | 0.727 | 0.104 |
| Reasoning problem solving | 38.67±10.56 | 42.07±12.46 | -1.010 | 0.317 | 0.294 |
| Social cognition | 38.94±9.30 | 39.24±12.83 | -0.089 | 0.920 | 0.026 |
| Total score | 35.33±11.82 | 38.15±13.47 | -0.764 | 0.448 | 0.222 |

The seven subdomain T-scores and composite T-score of MCCB were not significantly different in SCZ patients with and without clozapine treatment.

Table 2. T cell composition

| % | Clozapine  (n=20) | Non_Clozapine  (n=45) | *t* | *p* | *Cohen's d* |
| --- | --- | --- | --- | --- | --- |
| CD4+ cells % |  |  |  |  |  |
| CD4+ | 53.66±10.85 | 53.94±13.18 | -0.065 | 0.948 | 0.12 |
| CD4+CD45RA+ | 37.70±13.66 | 42.50±12.84 | -1.075 | 0.289 | 0.13 |
| CD4+CD45RO+ | 62.25±13.65 | 57.40±12.76 | 1.092 | 0.282 | 0.13 |
| CD4+CD95+ | 73.50±12.59 | 71.42±11.17 | 0.527 | 0.601 | 0.11 |
| CD4+CD45RA+CD95+ | 20.94±6.32 | 24.65±5.98 | -1.784 | 0.082 | 0.60 |
| CD4+CD45RO+CD95+ | 57.25±13.64 | 52.21±14.36 | 1.041 | 0.304 | 0.14 |
| CD8+ cells % |  |  |  |  |  |
| CD8+ | 39.09±8.33 | 35.94±10.24 | 0.946 | 0.350 | 0.10 |
| CD8+CD45RA+ | 51.39±20.50 | 62.07±14.83 | -1.885 | 0.067 | 0.16 |
| CD8+CD45RO+ | 48.50±20.59 | 37.83±14.84 | 1.877 | 0.068 | 0.16 |
| CD8+CD95+ | 84.55±12.61 | 84.35±10.87 | 0.051 | 0.959 | 0.11 |
| CD8+CD45RA+CD95+ | 37.80±17.51 | 48.40±15.58 | -1.924 | 0.062 | 0.16 |
| CD8+CD45RO+CD95+ | 45.02±20.28 | 33.93±14.21 | 2.013 | **0.051** | 0.16 |
| CD4+/CD8+ | 1.54±0.70 | 1.76±0.90 | -0.737 | 0.466 | 0.85 |

The percentages of naïve T cell subsets (CD4+CD45RA+, CD8+CD45RA+, CD4+CD45RA+CD95+, and CD8+CD45RA+CD95+ T cell subsets) and memory T cell subsets (CD4+CD45RO+, CD4+CD95+, CD4+CD45RO+CD95+, CD8+CD45RO+, CD8+CD45RO+CD95+ T cell subsets) were not significantly different in SCZ patients with and without clozapine treatment. Only the percentage of senescent CD8+CD45RO+CD95+ T cells show a possible trend toward statistically significant higher level in those with clozapine than those without.

Table 3. Intracellular cytokine levels in CD8+CD45RA+/CD45RO+CD95+ T cell subsets

| % | Clozapine  (n=20) | Non_Clozapine  (n=45) | *t* | *p* | *Cohen's d* |
| --- | --- | --- | --- | --- | --- |
| CD8+CD45RA+CD95+ |  |  |  |  |  |
| IL-1β | 9.67±4.48 | 9.28±4.78 | 0.247 | 0.806 | 0.47 |
| IL-6 | 24.95±10.11 | 29.88±18.93 | -0.851 | 0.400 | 0.16 |
| TNF-α | 10.51±7.85 | 7.51±6.45 | 1.282 | 0.207 | 0.06 |
| INF-γ | 3.73±2.39 | 3.08±2.27 | 0.839 | 0.407 | 0.02 |
| CD8+CD45RO+CD95+ |  |  |  |  |  |
| IL-1β | 10.54±4.03 | 9.38±4.62 | 0.751 | 0.457 | 0.04 |
| IL-6 | 35.60±13.23 | 38.05±21.54 | -0.366 | 0.716 | 0.19 |
| TNF-α | 22.61±11.23 | 18.38±10.68 | 1.141 | 0.260 | 0.10 |
| INF-γ | 10.61±3.77 | 8.78±5.07 | 1.131 | 0.265 | 0.04 |

The level of cytokines derived from CD8+CD45RA+CD95+ and CD8+CD45RO+CD95+ T cell subsets were not significantly different in SCZ patients with and without clozapine treatment.

Table 4. Comparison of subcortical volumes and cortical thicknesses between groups

|  | Clozapine  (n=20) | Non_Clozapine  (n=45) | *t* | *p* | *Cohen's d* | |
| --- | --- | --- | --- | --- | --- | --- |
| ICV (cm^3^) | 1591.12±18.24 | 1587.04±15.19 | 0.094 | 0.925 | | 0.029 |
| *Subcortical volume (cm^3^)* |  |  |  |  | |  |
| Lateral ventricle _L | 15.56±9.75 | 13.30±6.21 | 0.981 | 0.332 | | 0.30 |
| Lateral ventricle _R | 13.53±9.21 | 11.23±4.79 | 1.156 | 0.254 | | 0.35 |
| 3^rd^ ventricle | 2.04±0.86 | 1.73±0.68 | 1.151 | 0.255 | | 0.35 |
| 4^th^ ventricle | 2.07±0.73 | 2.09±0.51 | -0.120 | 0.905 | | 0.03 |
| CP_L | 0.60±0.20 | 0.59±0.19 | 0.118 | 0.907 | | 0.03 |
| CP_R | 0.59±0.22 | 0.64±0.19 | -0.806 | 0.425 | | 0.25 |
| *Cortical thickness (mm)* |  |  |  |  | |  |
| Thickness _L | 2.44±0.07 | 2.47±0.09 | -1.160 | 0.252 | | -0.360 |
| Thickness _R | 2.44±0.06 | 2.45±0.09 | -0.144 | 0.886 | | -0.045 |
| IPL_L | 2.40±0.09 | 2.44±0.17 | -0.654 | 0.516 | | -0.20 |
| IPL_R | 2.37±0.21 | 2.33±0.21 | 0.474 | 0.638 | | 0.14 |

The subcortical volumes and cortical thicknesses were not significantly different in SCZ patients with and without clozapine treatment.

***(3) The Comparison between Patients with and without Olanzapine.***

Table 1. MCCB scores

|  | Olanzapine (n=29) | Non_ Olanzapine (n=36) | *t* | *p* | *Cohen's d* |
| --- | --- | --- | --- | --- | --- |
| Speed of processing | 41.44±15.83 | 45.94±14.91 | -1.121 | 0.267 | 0.293 |
| Attention/vigilance | 32.22±10.41 | 36.29±10.47 | -1.480 | 0.145 | 0.389 |
| Verbal learning | 46.22±17.67 | 44.25±15.87 | 0.451 | 0.653 | 0.117 |
| Visual learning | 39.37±12.77 | 41.91±11.18 | -0.813 | 0.420 | 0.211 |
| Working memory | 37.22±15.57 | 38.94±14.71 | -0.434 | 0.666 | 0.113 |
| Reasoning problem solving | 40.15±12.98 | 41.78±11.11 | -0.521 | 0.605 | 0.135 |
| Social cognition | 37.04±12.78 | 40.94±10.74 | -1.274 | 0.208 | 0.330 |
| Total score | 35.41±13.80 | 38.90±12.15 | -1.026 | 0.309 | 0.268 |

The seven domain T-scores and composite T-score of MCCB were not significantly different in SCZ patients with and without olanzapine treatment.

Table 2. T cell composition

| % | Olanzapine (n=29) | Non_ Olanzapine (n=36) | *t* | *p* | *Cohen's d* |
| --- | --- | --- | --- | --- | --- |
| CD4+ cells % |  |  |  |  |  |
| CD4+ | 55.86±13.64 | 57.84±12.32 | -0.570 | 0.571 | 0.12 |
| CD4+CD45RA+ | 38.79±12.44 | 43.05±13.58 | -1.051 | 0.300 | 0.13 |
| CD4+CD45RO+ | 61.05±12.36 | 56.91±13.57 | 1.023 | 0.312 | 0.13 |
| CD4+CD95+ | 74.62±11.11 | 69.86±11.57 | 1.351 | 0.184 | 0.11 |
| CD4+CD45RA+CD95+ | 24.57±6.42 | 22.78±6.10 | 0.925 | 0.361 | 0.06 |
| CD4+CD45RO+CD95+ | 56.83±12.51 | 51.03±15.18 | 1.331 | 0.191 | 0.14 |
| CD8+ cells % |  |  |  |  |  |
| CD8+ | 37.81±10.83 | 36.00±10.37 | 0.550 | 0.586 | 0.11 |
| CD8+CD45RA+ | 58.47±17.32 | 59.47±17.28 | -0.186 | 0.853 | 0.17 |
| CD8+CD45RO+ | 41.41±17.34 | 40.45±17.33 | 0.179 | 0.859 | 0.17 |
| CD8+CD95+ | 83.06±10.24 | 79.13±11.65 | 1.152 | 0.256 | 0.11 |
| CD8+CD45RA+CD95+ | 48.75±19.10 | 42.59±14.17 | 1.198 | 0.238 | 0.16 |
| CD8+CD45RO+CD95+ | 37.64±17.26 | 36.65±16.59 | 0.188 | 0.852 | 0.16 |
| CD4+/CD8+ | 1.58±0.89 | 1.79±0.81 | -0.809 | 0.423 | 0.849 |

The percentages of naïve T cell subsets (CD4+CD45RA+, CD8+CD45RA+, CD4+CD45RA+CD95+, and CD8+CD45RA+CD95+ T cell subsets) and memory T cell subsets (CD4+CD45RO+, CD4+CD95+, CD4+CD45RO+CD95+, CD8+CD45RO+, CD8+CD45RO+CD95+ T cell subsets) were not significantly different in SCZ patients with and without olanzapine treatment.

Table 3. Intracellular cytokine levels in CD8+CD45RA+/CD45RO+CD95+ T cell subsets

| % | Olanzapine (n=29) | Non_ Olanzapine (n=36) | *t* | *P* | *Cohen's d* |
| --- | --- | --- | --- | --- | --- |
| CD8+CD45RA+CD95+ |  |  |  |  |  |
| IL-1β | 8.92±5.31 | 9.77±4.10 | -0.585 | 0.562 | 0.47 |
| IL-6 | 28.01±16.76 | 28.86±17.40 | -0.159 | 0.874 | 0.17 |
| TNF-α | 8.81±7.14 | 10.75±7.74 | -0.834 | 0.409 | 0.07 |
| INF-γ | 2.90±2.64 | 3.58±1.98 | -0.959 | 0.343 | 0.02 |
| CD8+CD45RO+CD95+ |  |  |  |  |  |
| IL-1β | 9.00±4.86 | 10.31±4.08 | -0.947 | 0.349 | 0.04 |
| IL-6 | 36.01±19.50 | 38.46±19.68 | -0.402 | 0.690 | 0.19 |
| TNF-α | 17.95±11.29 | 20.94±10.57 | -0.884 | 0.382 | 0.10 |
| INF-γ | 8.87±6.01 | 9.66±3.54 | -0.530 | 0.599 | 0.04 |

The level of cytokines derived from CD8+CD45RA+CD95+ and CD8+CD45RO+CD95+ T cell subsets were not significantly different in SCZ patients with and without olanzapine treatment.

Table 4. Comparison of subcortical volumes and cortical thicknesses between groups

|  | Olanzapine (n=29) | Non_ Olanzapine (n=36) | *t* | *p* | *Cohen's d* | |
| --- | --- | --- | --- | --- | --- | --- |
| ICV (cm^3^) | 1594.65±16.45 | 1584.24±15.95 | 0.222 | 0.826 | | 0.064 |
| *Subcortical volume (cm^3^)* |  |  |  |  | |  |
| Lateral ventricle _L | 13.95±0.56 | 14.02±8.56 | -0.023 | 0.975 | | -0.009 |
| Lateral ventricle _R | 12.09±4.59 | 11.81±7.5 | 0.146 | 0.885 | | 0.42 |
| 3^rd^ ventricle | 1.90±0.72 | 1.75±0.77 | 0.671 | 0.506 | | 0.195 |
| 4^th^ ventricle | 2.04±0.42 | 2.11±0.68 | -0.414 | 0.680 | | -0.120 |
| CP_L | 0.65±0.20 | 0.55±0.18 | 1.817 | 0.076 | | 0.528 |
| CP_R | 0.67±0.16 | 0.58±0.19 | 1.686 | 0.099 | | 0.506 |
| *Cortical thickness (mm)* |  |  |  |  | |  |
| Thickness _L | 2.45±0.10 | 2.46±0.07 | -0.473 | 0.638 | | -0.138 |
| Thickness _R | 2.43±0.10 | 2.45±0.07 | -0.792 | 0.432 | | -0.230 |
| IPL_L | 2.43±0.18 | 2.43±0.13 | 0.024 | 0.981 | | 0.007 |
| IPL_R | 2.32±0.22 | 2.36±0.21 | -0.801 | 0.427 | | -0.233 |

The subcortical volumes and cortical thicknesses were not significantly different in SCZ patients with and without olanzapine treatment.

***(4) Psychopharmacological Treatment Profiles***

The psychopharmacological treatment profiles of 65 SCZ patients were categorized as follows:

①Monotherapy with antipsychotics (n=35):​

Haloperidol (n=1), perphenazine (n=1), paliperidone (n=5), olanzapine (n=13), clozapine (n=9), risperidone (n=5), blonanserin (n=1).

​**​**② Dual antipsychotic therapy (n=20):​​

Olanzapine + haloperidol (n=2), paliperidone + sulpiride (n=1), clozapine + olanzapine (n=1), olanzapine + risperidone (n=2), olanzapine + amisulpride (n=2), amisulpride + aripiprazole (n=1), clozapine + risperidone (n=1), amisulpride + perospirone (n=1), olanzapine + aripiprazole (n=3), clozapine + paliperidone (n=2), clozapine + aripiprazole (n=3), clozapine + blonanserin (n=1).

③ Triple antipsychotic therapy (n=1):​​

Clozapine + aripiprazole + olanzapine (n=1).

​**​**④ Antipsychotics combined with mood stabilizers (n=7):​​

Haloperidol + olanzapine + sodium valproate (n=1), olanzapine + sodium valproate (n=1), clozapine + quetiapine + sodium valproate (n=1), clozapine + lithium carbonate (n=1), paliperidone + olanzapine + sodium valproate (n=1), olanzapine + lithium carbonate (n=1), risperidone + lithium carbonate (n=1).

​**​**⑤ Antipsychotics combined with antidepressants (n=2):​​

Olanzapine + sulpiride + duloxetine (n=1), lurasidone + escitalopram (n=1).

The number of subjects administered for each psychopharmacological agent and the corresponding dosage were provided in the following table.

Table. The number of subjects administered each psychopharmacological agent and th2corresponding dosage

| FGAs | n | Dosage  (mg)  M (SD) | SGAs | n | Dosage  (mg)  M (SD) | Mood  Stabilizers | n | Dosage  (mg)  M (SD) | Antidepressants | n | Dosage  (mg)  M (SD) |
| --- | --- | --- | --- | --- | --- | --- | --- | --- | --- | --- | --- |
| Haloperidol | 4 | 15.5​0  (3.57​) | Amisulpride | 40 | 600.00  (163.30) | Sodium  Valproate | 4 | 562.50  ​(108.25​) | Duloxetine | 1 | 60.00 |
| Perphenazine | 1 | 18.00 | Aripiprazole | 8 | 15.50  (5.59) | Lithium  Carbonate | 3 | 466.67​  (124.72) | Escitalopram | 1 | 5.00 |
| Sulpiride | 2 | 600.00  (200.0) | Blonanserin | 2 | 16.00  (8.00) |  |  |  |  |  |  |
|  |  |  | Quetiapine | 1 | 500.00 |  |  |  |  |  |  |
|  |  |  | Olanzapine | 29 | 15.34  (4.68) |  |  |  |  |  |  |
|  |  |  | Paliperidone | 9 | 7.00  (2.00) |  |  |  |  |  |  |
|  |  |  | Risperidone | 9 | 4.44  (1.17) |  |  |  |  |  |  |
|  |  |  | Perospirone | 1 | 8.00 |  |  |  |  |  |  |
|  |  |  | Lurasidone | 1 | 40.00 |  |  |  |  |  |  |
|  |  |  | Clozapine | 20 | 182.30  (133.21) |  |  |  |  |  |  |

FGA, first generation antipsychotics; SGA, second generation antipsychotics.
